# Raman spectroscopic cellomics for the detection of SARS‐CoV‐2‐associated neutrophil activation after TNF‐α stimulation

**DOI:** 10.1002/ctm2.1139

**Published:** 2022-12-19

**Authors:** Aikaterini Pistiki, Franziska Hornung, Anja Silge, Anuradha Ramoji, Oleg Ryabchykov, Thomas W. Bocklitz, Karina Weber, Bettina Löffler, Jürgen Popp, Stefanie Deinhardt‐Emmer

**Affiliations:** ^1^ Friedrich Schiller University Jena, Institute of Physical Chemistry and Abbe Center of Photonics Jena Germany; ^2^ InfectoGnostics Research Campus Jena Center for Applied Research Jena Germany; ^3^ Leibniz Institute of Photonic Technology Jena Germany; ^4^ Institute of Medical Microbiology, Jena University Hospital Jena Germany

Dear Editor,

Coronavirus disease 2019 (COVID‐19) demonstrates that respiratory viruses can induce severe lung damage and systemic inflammation with strongly activated immune response. Since new diagnostic strategies are required to identify critical illness, we studied the course of viral infections by using immune cells. Here, phenotypic studies can reveal changes in cell structure after infection or inflammatory stimulation.^1^ Patients suffering from systemic infections could benefit from phenotypic characterization of innate immune cells. Neutrophil granulocytes represent an important part of the innate immune response, involved in combatting viral pathogens.^2^

The detection technology based on Raman spectroscopy (RS) has been applied in virus detection.^3^ High throughput screening RS (HTS‐RS) represents an innovative strategy for direct and non‐destructive detection of changes in the composition and phenotype of the white blood cells.^4^, ^5^, ^6^, ^7^ The principle of this technique is to irradiate a cell with laser light for a few seconds and record the inelastically scattered light as a spectrum.^4^ Compared to traditional flow cytometry, this spectrum displays the sample's label‐free molecular signature, which is susceptible to change due to biological stimulations.^5^, ^6^, ^7^ Machine learning approaches are applied to transfer the spectral into meaningful biological information.^8^

It is well‐established that neutrophil granulocytes require pre‐stimulation to induce their inflammatory capacity.^1^ In our study, we are using RS to examine the cell response of non‐activated, tumor necrosis factor‐alpha (TNF‐α)‐ and LPS‐activated neutrophils to SARS‐CoV‐2 infection. The influence of the interaction time between virus and cell on the spectral phenotype of neutrophils and on the secretion level of chemokines/cytokines was investigated (Figure 1).

**FIGURE 1:**
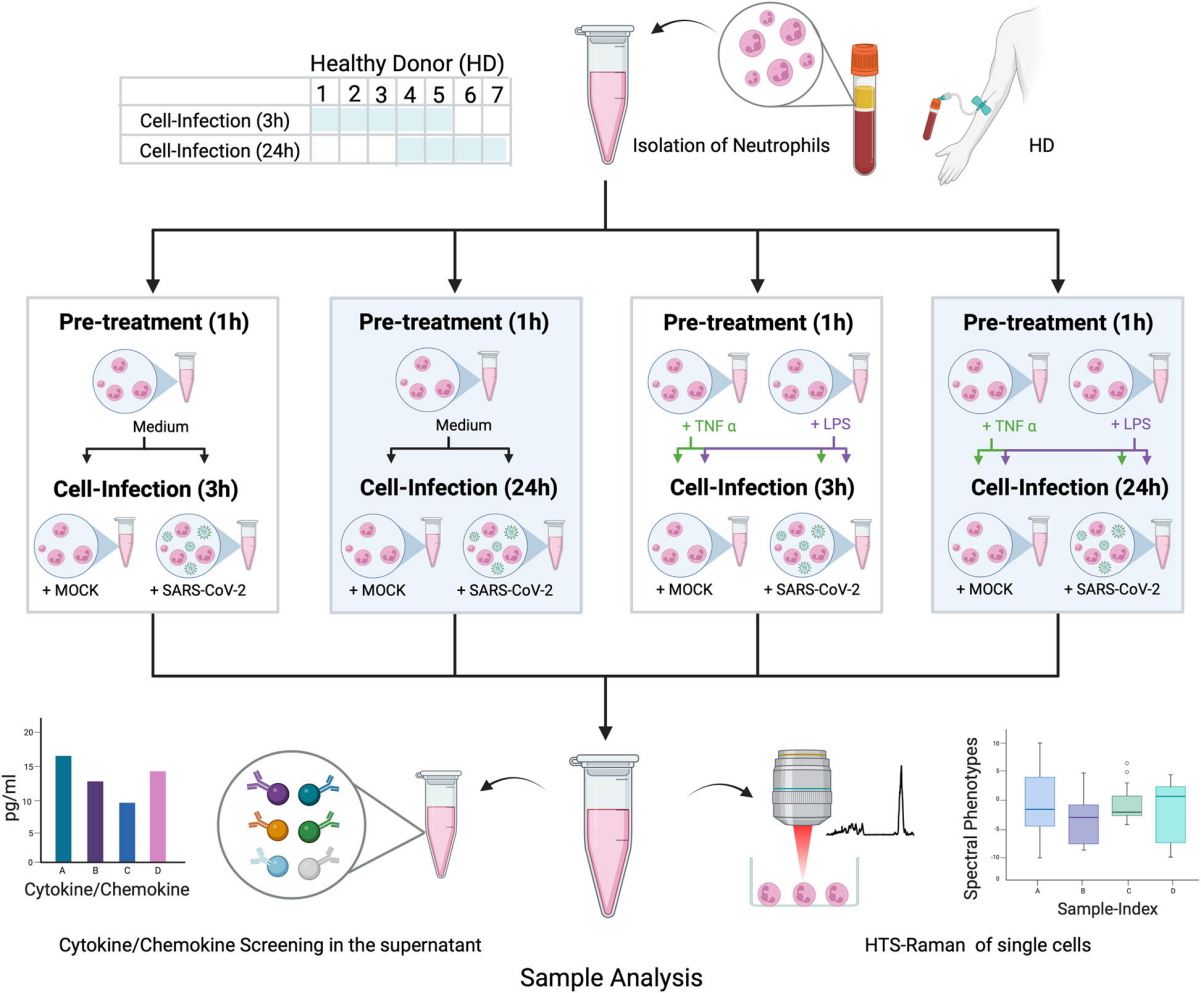
Study flow chart indicating isolation, pre‐treatment, infection and sample analysis. Blood was withdrawn from healthy donors (HD) numbers 1–5 for experiments with 3 h neutrophil‐virus interaction and from HD numbers 4–7 for experiments with 3‐h neutrophil‐virus interaction. In a first step, neutrophils were investigated without any activation by typical chemo‐ or cytokines (pre‐treatment = medium). In the second part, cells were pre‐treated before SARS‐CoV‐2 infection with the specific neutrophil chemoattractant tumor necrosis factor‐alpha (TNF‐α) or lipopolysaccharides (LPS) for 1 h each. Mock and SARS‐CoV‐2 infection were performed independently for each pre‐treatment (indicated by the green and purple arrows in case of pre‐treatment with TNF‐α and LPS in Figure 1). Raman spectra from all datasets collected. Technological specifications of the HTS‐Raman set up are published previously (3, 4). Figure was created with BioRender.com

A partial least‐squares discriminant analysis (PLS‐DA) was applied to differentiate between Raman data of the infected and control cells. To estimate prediction performance, a leave‐one‐donor‐out cross‐validation (LODO‐CV) method was used. Prediction values are plotted against the true class labels and summarized in box plots (Figure 2A). The balanced accuracy (mean over sensitivity and specificity on the sample level) for mock versus SARS‐CoV‐2 infected neutrophils was 40 % after 3 h and 50 % after 24 h of virus‐cell interaction. Subsequently, the phenotype recorded in the Raman spectra does not change.

**FIGURE 2:**
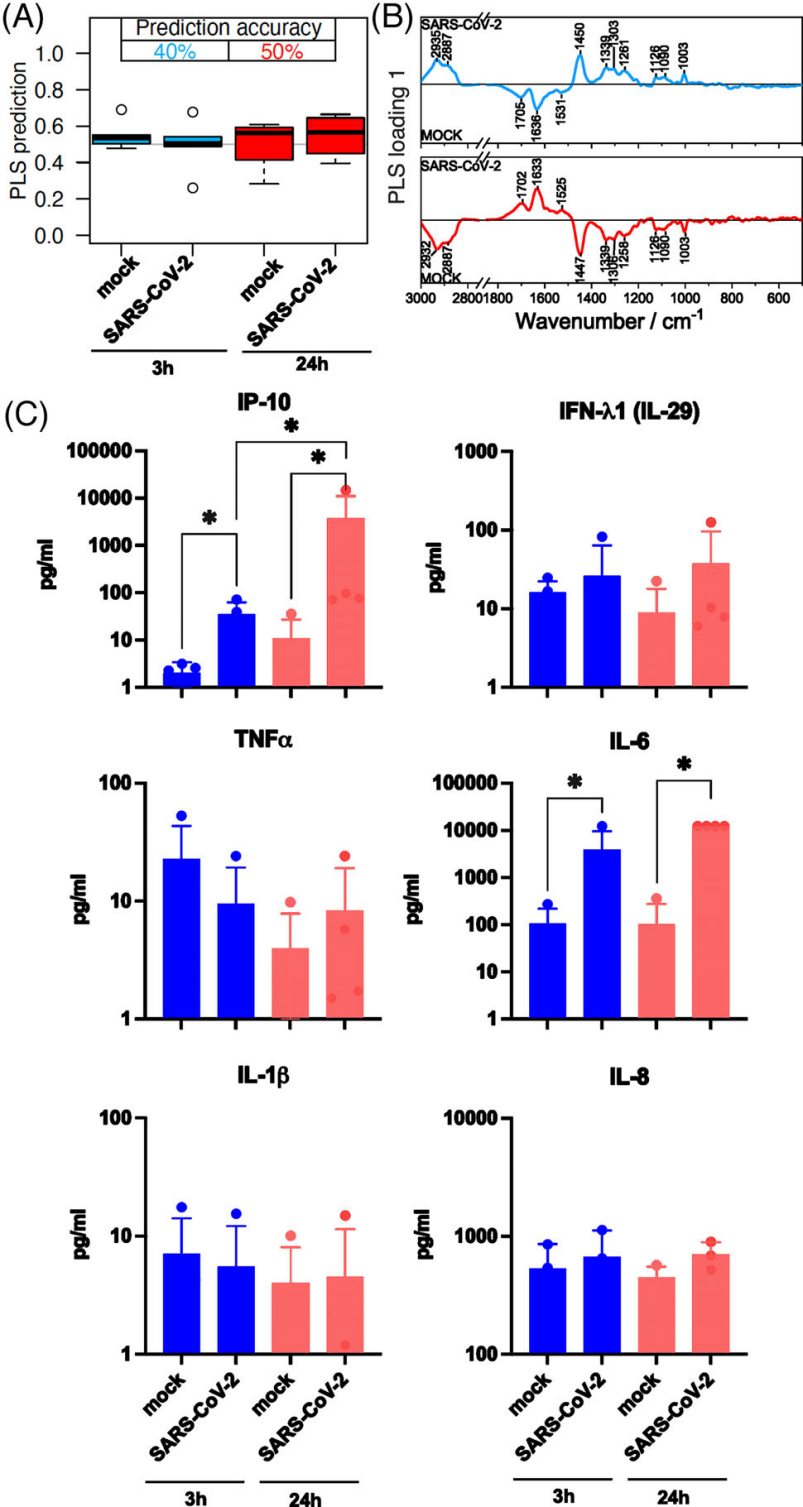
Comparison of neutrophil granulocytes after 3 and 24 h mock and SARS‐CoV‐2 infection. (A) The box plot summarizes the prediction results of the partial least‐squares discriminant analysis (PLS‐DA) model mock versus SARS‐CoV‐2 based on the Raman spectral phenotypes of the cells. Samples with values below 0.5 were predicted as non‐infected cells (not challenged by the virus), and samples with values above 0.5 were predicted as infected cells. Blue boxes reveal the results after a 3‐h infection period; the balanced accuracy of the model is highlighted in blue. Red boxes summarize the results of the 24 h model, and the balanced accuracy is highlighted in red. (B) Loading of the PLS model provide insights which Raman spectral features in the model revealed minor differences between the mock and SARS‐CoV‐2 classes. (C) Bar plots present the mean of all individual values and show the indicated cytokine and chemokine levels measured in the cell supernatant 3 h (blue bars) and 24 h (red bars) p.i. (post infection). Statistical significance was determined via two‐tailed Mann–Whitney test, *p < 0.05. The data that support the findings of this study are available from the corresponding author upon reasonable request.

The PLS loadings (Figure 2B) reveal the undirected spectral variations within the Raman data. Main contributions are assigned to the Amide I band around 1660 cm^−1^ and the Amide III band around 1240 cm^−1^.^9^ The functional amino acid tryptophan typically contributes to the signal around 1550 cm^−1^.^9^ The quite intense signal around 1450 cm^−1^ is due to the CH_2_ deformation vibration shared by lipids and proteins.^9^ The region 1200–1300 cm^−1^ is characterized by highly mixed vibrational bands involving the amide III, contributions from the amino acid phenylalanine and tyrosine and nucleic acids.^9^ The C‐N stretch vibration of proteins appears at 1130 cm^−1^.^9^

To verify a successful infection, we determined the production of inflammatory cytokines after 3 h (Figure 2C, blue bars) and 24 h (Figure 2C, red bars) virus‐cell interaction in the supernatant of the cell culture. Here, SARS‐CoV‐2 infection leads to significant increase of the Interferon‐gamma induced protein 10 (IP‐10), a highly relevant virus‐induced chemokine. Also, the Interleukin (IL)‐6 level shows significantly higher levels in the supernatant of infected cells. After 24 h, the IP‐10 level increased about 100‐fold and the IL‐6 level increased about 10‐fold in the SARS‐CoV‐2 infected cells compared to the 3‐h infection period.

Physiological neutrophil activation and recruitment is mimicked by pre‐treatment with TNF‐α or LPS to simulate virus infection.^1^, ^10^ In the group of TNF‐α pre‐stimulated cells, classification accuracy achieved 60 %. A prominent effect could be observed for LPS pre‐stimulated cells (accuracy 80 %). The loadings show similar patterns for TNF‐α and LPS (Figure 3A). Significant differences could be detected in the supernatant for IP‐10 and IL‐6 for both pre‐stimulations (Figure 3C). The levels were comparable with the 3 h infection period without pre‐stimulation (Figure 2C blue bars).

**FIGURE 3:**
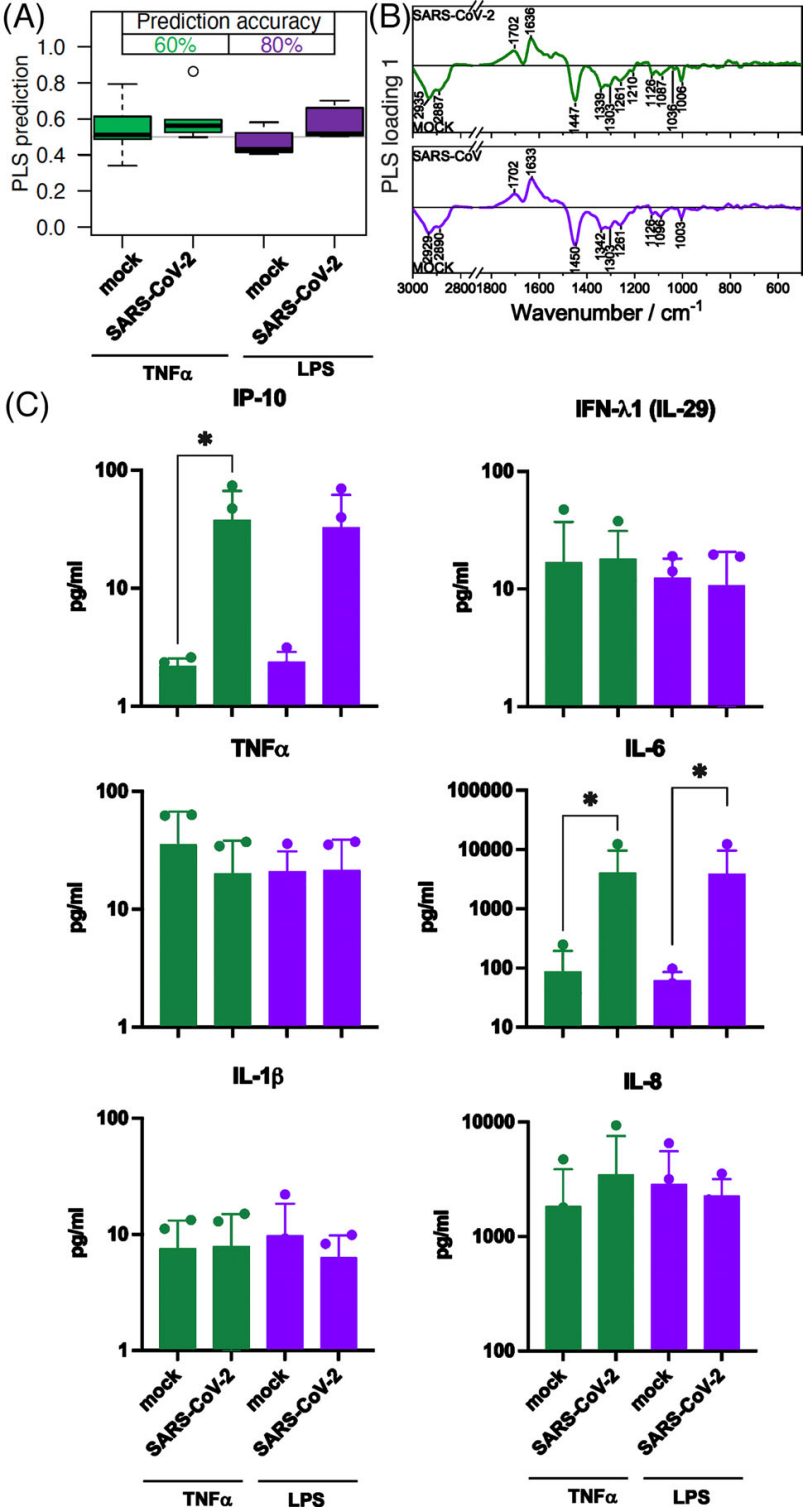
Comparison of neutrophil granulocytes after 1‐h pre‐treatment with TNF‐α and lipopolysaccharides (LPS) followed by a 3‐h mock or SARS‐CoV‐2 infection. (A) The box plot summarizes the prediction results of the model mock versus SARS‐CoV‐2 based on the Raman spectral phenotypes of the cells. Green boxes reveal the results after a TNF‐α pre‐treatment and 3‐h infection period; the balanced accuracy of the model is highlighted in green. Purple boxes summarize the results of the LPS pre‐treatment and 3‐h infection period model, and the balanced accuracy is highlighted in purple. (B) Loading of the partial least‐squares (PLS) model provide insights which Raman spectral features in the model revealed differences between the mock and SARS‐CoV‐2 classes. (C) Bar plots present the mean of all individual values and show the indicated cytokine and chemokine levels measured in the cell supernatant after TNF‐α pre‐treatment (green bars) and LPS pre‐treatment (green bars) and 3‐of infection. Statistical significance was determined via two‐tailed Mann Whitney test *p < 0.05. The data that support the findings of this study are available from the corresponding author upon reasonable request. TNF‐α, tumor necrosis factor‐alpha.

After 24 h infection, classification accuracy of TNF‐α pre‐stimulated cells increases to 75 % (green boxes Figure 4A). The effect of the LPS pre‐stimulation increases slightly and achieved 87.5 % accuracy (purple boxes Figure 4A). The concentration of proinflammatory cytokines increases in intensity after SARS‐CoV‐2 infection and subsequent stimulation for 24 h (Figure 4C). No effect of the pre‐treatment itself on the differentiation of the Raman data of the investigated cells was observed.

**FIGURE 4:**
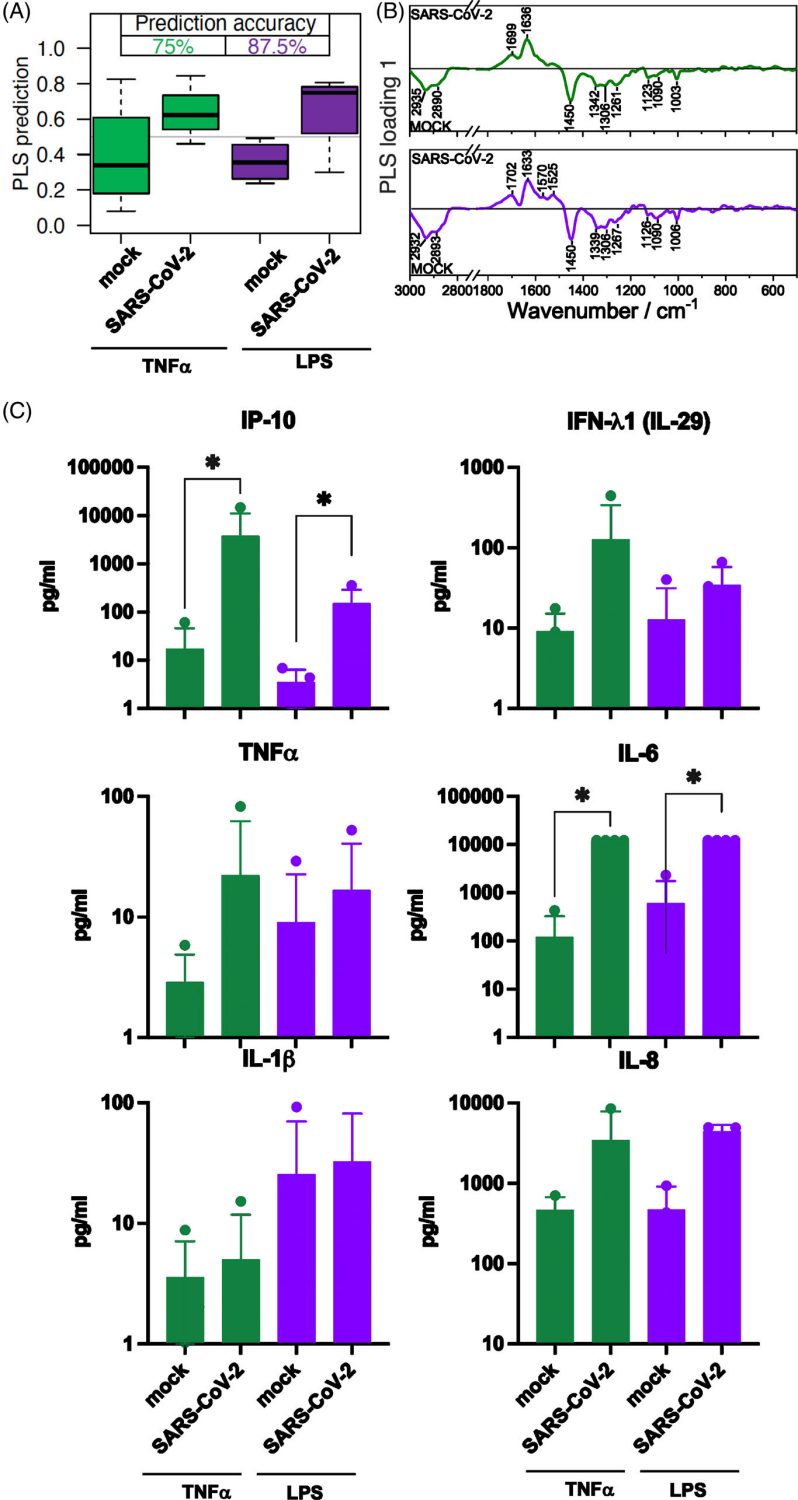
Comparison of neutrophil granulocytes after 1 h pre‐treatment with TNF‐α and lipopolysaccharides (LPS) followed by a 24‐h mock or SARS‐CoV‐2 infection. (A) The box plot summarizes the prediction results of the model mock versus SARS‐CoV‐2 based on the Raman spectral phenotypes of the cells. Green boxes reveal the results after a TNF‐α pre‐treatment and 24‐h infection period, the balanced accuracy of the model is highlighted in green. Purple boxes summarize the results of the LPS pre‐treatment and 24‐h infection period model, and the balanced accuracy is highlighted in purple. (B) Loading of the partial least‐squares (PLS) model provide insights which Raman spectral features in the model revealed differences between the mock and SARS‐CoV‐2 classes. (C) Bar plots present the mean of all individual values and show the indicated cytokine and chemokine levels measured in the cell supernatant after TNF‐α pre‐treatment (green bars) and LPS pre‐treatment (green bars) and 24‐h infection. Statistical significance was determined via two‐tailed Mann–Whitney test *p < 0.05. The data that support the findings of this study are available from the corresponding author upon reasonable request.

Our study demonstrates that the Raman spectral immune phenotype can be used to determine pre‐activated neutrophil granulocytes challenged with SARS‐CoV‐2. The increased neutrophil count has been observed in patients with severe COVID‐19.^1^ The neutrophil response to SARS‐CoV‐2 infection occurs through immune activation and results in various phenotypic changes like degranulation and the release of pro‐inflammatory cytokines.^1^, ^2^ Variations associated with the SARS‐CoV‐2 ‐ neutrophil interaction are pronounced in the wavenumber region of the Amide I band and between 1550  and 1580 cm^−1^, which gives an indication of changes in the tryptophan homeostasis of the neutrophils. The tryptophan metabolism pathway plays a crucial role in inflammation and immune tolerance captured in Raman spectra.^7^

Our findings indicate a respond of neutrophil granulocytes to SARS‐CoV‐2 by producing antiviral and inflammatory substances, including IP‐10 and IL‐6, identified as an independent predictor for disease progression.^1^, ^2^ In addition, pre‐stimulation by TNF‐ α and LPS results in increased expression of the measured cytokines. Since LPS is a strong stimulant in neutrophil granulocytes, it was used as a positive control.^1^ Neutrophile number and activation have beneficial and detrimental effects during viral infection.^1^

In our study, phenotypic change of neutrophil granulocytes was detectable in response to pre‐stimuli accompanied by SARS‐CoV‐2 infection using HTS RS. The data obtained correlate strongly with the expression of proinflammatory cytokines and show that RS is an excellent method to characterize the phenotypic response of immune cells.

Comprehensive characterization of neutrophil's spectral immunotypes following SARS‐CoV‐2 contact provides the fundamental to make Raman spectral cell data a rapidly available predictor of important clinical features associated with COVID‐19.

1. Pre‐activated neutrophil granulocytes challenged with SARS‐CoV‐2 are identified using label‐free high‐throughput Raman screening.
2. The obtained data strongly correlate with the expression of proinflammatory cytokines, demonstrating that RS combined with machine learning is an excellent method for characterizing the phenotypic response of immune cells.
3. The findings show that neutrophil granulocytes respond to SARS‐CoV‐2 infection by producing antiviral and inflammatory substances such as IP‐10 and IL‐6, which have been linked to COVID‐19 severity and identified as an independent predictor of disease progression.

## CONFLICT OF INTEREST

The authors declare that they have no conflict of interest.
